# Do current family history-based genetic testing guidelines contribute to breast cancer health inequities?

**DOI:** 10.1038/s41523-022-00391-4

**Published:** 2022-03-22

**Authors:** Samantha H. Jakuboski, Jasmine A. McDonald, Mary Beth Terry

**Affiliations:** 1 Stritch School of Medicine, Loyola University Chicago, Maywood, IL 60153 USA; 2 Mailman School of Public Health, Columbia University Irving Medical Center, New York, NY 10032 USA; 3 Herbert Irving Comprehensive Cancer Center, Columbia University Irving Medical Center, New York, NY 10032 USA

**Keywords:** Cancer epidemiology, Breast cancer

## Abstract

Prior to the recommended age for population-based breast cancer screening by mammography, which ranges from 40−50 years depending on guidelines, the main way to identify higher risk women for earlier breast cancer (BC) screening to improve outcomes and discuss targeted chemoprevention is through specific clinical guidelines which are largely based on family history of breast cancer and known mutations in breast cancer susceptibility genes. The annual percent change (APC) in early-onset BC continues to rise, with the higher early-onset cancer burden and mortality continuing to be seen in non-Hispanic black (NHB) women compared to non-Hispanic white (NHW) women. Coupled with the increasing incidence overall as well as the lower percent of BC family history reported in NHB women compared with that of NHW women means that continued reliance on guidelines to identify women for genetic screening and initiation of early BC screening based largely on family history could lead to even greater BC health inequities. The similarity in the prevalence of mutations in key BC susceptibility genes between NHB and NHW women contrasts sharply to the differences in age-specific incidence rates between NHB and NHW women, supporting that there must be environmental modifiers that are contributing to the increased incidence in NHB women. This reality further argues for identifying NHB women early in adulthood through genetic testing who may benefit from tailored BC risk-reduction programs and early BC screening.

## Early onset breast cancer continues to rise

The incidence of breast cancer (BC) in women under 55 continues to increase every year in the U.S., and the annual percent change (APC) is highest in women under 40 years^1^. BC is also increasing in women under age 50 years in most countries of the world^2^. Since population-based mammography is not available in many parts of the world and, where available, not recommended until after 45 or 50 years of age for most women, the clinical guidelines to identify higher risk women for earlier BC screening and targeted risk reduction are largely based on family history of cancer and/or known pathogenic variants (PV) in BC susceptibility genes. As family history cannot explain the increasing incidence trends, which are driven by changing environmental factors interacting with underlying susceptibility, current guidelines for identifying higher risk women are and will continue to be increasingly inadequate. Here we discuss the implication of maintaining family-based history guidelines as opposed to using population-based genetic testing to reduce BC health inequities and improve identification of higher risk women earlier to improve outcomes in the U.S. and globally.

### The inequitable BC burden

In the U.S. and globally, estrogen receptor (ER) negative BC continues to be associated with worse outcomes compared with ER positive BC. The burden of ER negative BC generally, and triple negative BC (TNBC, ER negative, progesterone receptor negative, and HER2 negative) specifically, is particularly high in non-Hispanic black (NHB) women, with approximately double the absolute TNBC rates in NHB compared to non-Hispanic (NHW) women^3^. TNBC, the most aggressive molecular subtype of BC, makes up approximately 11.3% of all breast cancers in the U.S, 19.5% of all breast cancers under 40 years of age, and 13.6% of breast cancers under the age of 55 years old (and an even higher proportion in countries without screening). Close to 70% of *BRCA1* associated BC is TNBC^3–5^. In contrast, up to 77% of *BRCA2* associated BC is ER+^4,6^. ER+ (including Luminal A and Luminal B) BC have been increasing in recent years. Rates of all molecular BC subtypes are similar, or much higher, as in the case of TNBC, in NHB women compared to NHW women under age 40 years^3^ (see Table 1 columns 2 and 3). In women 40 years and older, Luminal A BC is higher in NHW women but, similar to under age 40 years, other molecular subtypes are either similar or much higher, as is the case for TNBC between NHB and NHW^3^.

**Table 1:** Actual incidence rates and estimated rate differences for individuals without a family history between NHB and NHW women by age and breast cancer molecular subtype.

	NHB Incidence/100,000	NHW Incidence/100,000	Estimated incidence in NHB without family history (Per 100,000)	Estimated incidence in NHW without family history (Per 100,000)	Estimated Rate difference
Overall					
TNBC	38	19	31.2	12.5	18.7
Luminal A (HR+, HER2−)	112	138	94.1–96.9	107.1	−13 to −10.2
Luminal B (HR+, HER2+)	21	20	17.6–18.2	15.5	2.1–2.7
HER2+/enriched (HR−, HER2+)	10	8	8.4–8.7	6.2	2.2–2.5
<40 years					
TNBC	9.7	5.7	8	3.8	4.2
Luminal A	17.8	17.9	15–15.4	13.9	1.1–1.5
Luminal B	6.7	6.6	5.6–5.8	5.1	0.5–0.7
HER2+/enriched	2.6	2.4	2.2	1.9	0.3
40–54 years					
TNBC	34.6	17.5	28.4	11.6	16.8
Luminal A	84.4	113.3	70.9–73	87.9	−17 to −14.9
Luminal B	19.9	20.4	16.7–17.2	15.8	0.9-1.4
HER2+/enriched	10.4	7.6	8.7–9	5.9	2.8–3.1
55–69 years					
TNBC	52.6	25.1	43.1	16.6	26.5
Luminal A	164.3	207.9	138-142.1	161.3	−23.3 to −19.2
Luminal B	30.3	27.1	25.5-26.2	21	4.5-5.2
HER2+/enriched	14.9	11.3	12.5-12.9	8.8	3.7-4.1
70–84 years					
TNBC	49.6	27.8	43.1	16.6	22.4
Luminal A	214.1	268	138–142.1	161.3	−28.2 to −22.8
Luminal B	25.4	25.3	25.5–26.2	21	1.7–2.4
HER2+/enriched	14.7	9.9	12.5–12.9	8.8	4.6-5

FH% is 18%^40^, 13.5–16%^27,40^, 22.4%^27^, and 34%^41^ for NHB TNBC all ages, NHB FH for other BC subtypes all ages, NHW other subtypes all ages, and NHW TNBC all ages, respectively; Incidence data sources^3,39^.

With such grim statistics, how do we create greater health equity? Standard approaches will not suffice. Clinical guidelines continue to rely primarily on BC family history to identify higher risk women for earlier screenings and risk reduction interventions. Some of the cultural values and norms within black communities have been found to be associated with less communication regarding family cancer history, making family cancer history knowledge more limited^7–9^. In addition, even black women with PV who do meet clinical guidelines still do not receive genetic testing as part of their care^10^. Public health recommendations related to lifestyle factors, including breastfeeding recommendations, are important; yet, we know that established lifestyle risk factors can only explain a portion of BC incidence, particularly so for more aggressive BC. Lifestyle recommendations also lay the onus of mitigating these health inequities on the individual who may not reside in equitable conditions (e.g., workplaces without spaces for breastfeeding, lack of access to parks and healthy food). Here we propose one approach to consider by advocating for universal genetic testing within our health care system. Failure to identify higher-risk individuals, particularly before the age of population screening and in countries without population-based BC screening, will contribute to greater cancer health inequities, particularly with worse outcomes from more aggressive BC and later stage BC diagnoses.

### Why we should consider moving beyond family history-based guidelines

There is already a precedent to move beyond family history-based guidelines for women once they are diagnosed with TNBC. The National Comprehensive Cancer Network (NCCN) guidelines now support universal genetic testing of all women with TNBC, regardless of family history^11^. However, genetic testing of women diagnosed with other molecular subtypes is still based on a combination of age at diagnosis and family history. Current practice has been estimated to miss close to 50% of BC patients with PV^12–15^. There are important considerations in conducting genetic testing for patients with BC whose treatment may not be affected based on the presence of PV, like patients with TNBC^16^. However, a key argument of testing all women once diagnosed with BC includes the benefit of cascade testing of relatives; cost effectiveness modeling has supported such a strategy^17^.

Cascade testing, however, misses unaffected women without a family history, and as the incidence rates are increasing in women under 55 years in the U.S. and globally, it is important to consider other approaches for finding higher risk individuals. Although the prevalence estimates of PV are much lower in unaffected women compared to women once they have been diagnosed with BC^18^, the reality is that family history approaches for genetic testing also miss over 50% of BRCA1/2 PV in unaffected women^19,20^; missclassification of other BC PV is even higher^21^. As a result, women without a family history will generally not be offered genetic testing, and will not receive BC screening until the age of population-based mammography, which is usually age 50 or older for countries where it exists (45 now in the United States).

**Fig. 1:**
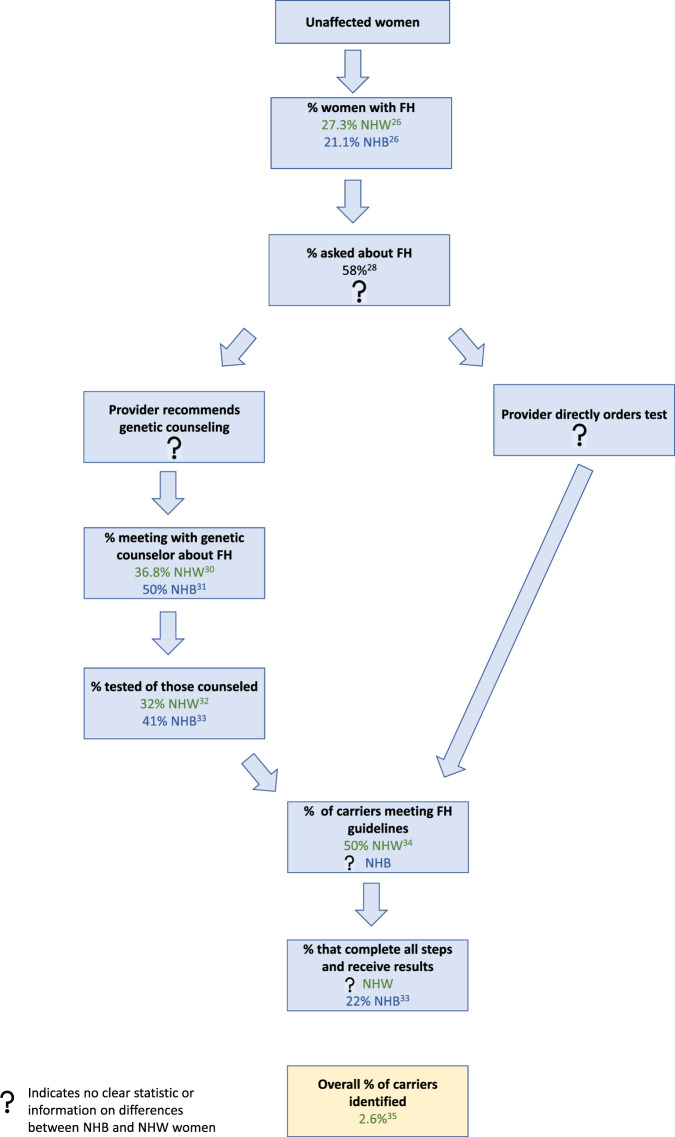
Multiple steps need to be taken using family history (FH) guidelines. There are many steps that need to be completed for an unaffected woman with a mutation in a BC susceptibility gene to receive BC genetic testing. These steps are outlined here, with data provided for both NHW and NHB women when available. Initially, let us assume an unaffected carrier meets cancer FH based criteria. The next step is that, she must be asked about her cancer FH by her health care provider (or volunteer the information). The health care provider must understand the current guidelines and either (a) recommend the woman for genetic counseling, (b) order the genetic test directly, or (c) decide that genetic testing is not needed (this branch is not shown). If a woman receives a recommendation for genetic counseling, she must then meet with the genetic counselor, undergo testing, and receive her results. Some of the articles cited above do not clarify if the participants are non-Hispanic. Abbreviations: NHW non-Hispanic white, NHB non-Hispanic black, FH family history.

Family history-based testing not only leads to many missed women who have PV in BC susceptibility genes across all women, but also it creates a differential between testing rates in black compared to white women. (The following discussion will classify patients as black, NHB, or African American, using the wording of the authors of the referenced studies). In Fig. 1, we outline ways that family history-based testing may affect who gets tested and who is offered earlier BC cancer screening. As illustrated, there are multiple steps and providers which all require coordination. At any step, women can fall off the path of genetic testing and earlier screening. A key challenge with the current guidelines is that not all women know their family history. The definition of family and the cultural norms and beliefs among black communities differ from white communities, which translates to differences in the collection of family cancer history^7,22–25^. Among some black communities, communication about family health history or family cancer history to a medical provider is rare^7–9^. Although providers emphasize the collection of family cancer history to inform prevention and screening, studies also suggest that communication about family cancer history can cause familial conflict and disruption^7^. Nonetheless, there are efforts emphasizing the importance of medical family history in the NHB community. However, we should ask: why do solutions for mitigating a historical inequity caused by macro-level societal factors lie within the individuals of that community, and not with society?

Of the women with knowledge of their family history, most estimates suggest that a much smaller percentage of black women report a BC family history compared with white women^26,27^. Providers are key in the process of recommending testing, yet one study of 1,700 unaffected women in the San Francisco Mammography Registry found that only 58% of women are asked about their cancer family history^28^. Once a physician elicits the family history, they can directly order a genetic test, refer the patient for genetic counseling, or decide not to recommend testing. While the data on the percent of unaffected women who are recommended genetic counseling are limited, data for women with BC shows that black BC patients are less likely to receive a recommendation for genetic testing than are white BC patients^29^. Of all women referred to counseling, studies support that only approximately 50% of women complete counseling, and an even smaller percentage of those counseled are actually tested^30–33^. Thus, even though public health initiatives that encourage the public to discuss, collect, and record family cancer history is important, reliance on family-based criteria will still result in missing many women with PV in breast cancer susceptibility genes^34^. One study suggested that only 2.6% of the general population with mutations may be identified with current approaches^35^.

### Similar prevalence of pathogenic variants in BC susceptibility genes

In addition to growing evidence supporting that family history-based criteria miss a large percentage of women with germline PV in BC susceptibility genes, several large and recent studies support the prevalence of these genes is similar across different groups based on race and ethnicity. For example, in a large population-based case control study of 3,946 black and 25,287 NHW women with BC, the prevalence of 12 breast cancer susceptibility genes was 5.7% and 5.1%, respectively^36^. Such similarities are also seen across BC subtypes, such as TNBC (10.3% and 10.2% for black and NHW women, respectively)^36^. Other studies support these similarities between race and ethnicity. For example, Couch and colleagues reported that germline multigene panel testing on a clinical cohort of 8,753 TNBC patients revealed no significant germline differences in the prevalence of PV (see Supplementary Table 2 from Reference^5^). PV prevalence for BRCA1/2 was 7.8% and 9.0% for Caucasian and African American (AA) women, respectively, and when expanding testing to include a panel of 21 BC susceptibility genes, PV prevalence increased to 14.0% and 14.6%, respectively^5^. Additionally, the magnitude of the association between PV in BC susceptibility genes and TNBC risk in AA women was reported to be higher than that reported for Caucasian women^5,21^. In a pooled analysis of several large epidemiologic studies in AA women, Palmer and colleagues also reported similarity in prevalence of germline PV in AA and white women with TNBC^37,38^. What is striking is that these studies had different ascertainment methods, but still supported similar percentages of BC PV between black and white women.

### Potential impact from family-based criteria

**Fig. 2:**
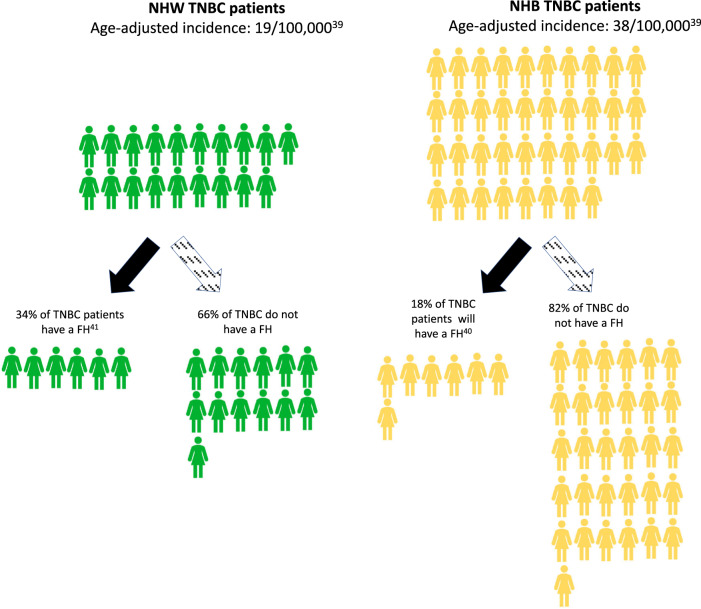
Family history guidelines lead to many missed women at risk for breast cancer. Potential rate difference between NHB and NHW women based on the estimated percent with family history of cancer for TNBC. The age-adjusted incidence for TNBC is 19 per 100,000 and 38 per 100,000 for NHW and NHB women, respectively. 34% and 18% of NHW and NHB TNBC patients, respectively, report a family-history of BC. Assuming a conservative 100% uptake in genetic counseling and testing, this translates into an estimated incidence rate difference between NHB and NHW women with TNBC without a family history of 31 versus 13 per 100,000. Abbreviations: NHW non-Hispanic white, NHB non-Hispanic black, TNBC triple negative breast cancer, FH family history.

In Fig. 2, we illustrate the potential rate difference between NHB and NHW women based on the estimated percent with family history of cancer for TNBC (Supplemental Fig. 1 illustrates the rate difference for women under 40 years old; Table 1 reports by molecular subtypes and ages). For example, the overall incidence rate for TNBC is 38 and 19 per 100,000 for NHB and NHW women, respectively^39^. Among NHB and NHW women diagnosed with TNBC, 18% and 34%, respectively, have a known family history of BC^40,41^. Thus, the estimated TNBC incidence for women without a reported family history is 31.2 versus 12.5 per 100,000 for NHB and NHW women, respectively. The same calculation for women diagnosed under 40 would be 8.0 and 3.8 per 100,000 for NHB and NHW women, respectively (see Table 1 and Supplemental Fig. 1). These estimates suggest a considerable number of NHB women who are diagnosed with TNBC would not have met family-history based screening criteria and would be less likely to undergo early BC screening. This is despite the fact that the percent with known PV is similar to that in NHW women. Table 1 shows the rate differences for women without a family history by molecular subtype. As mentioned above and worth reiterating, Table 1 illustrates that (with the exception of the Luminal A subtype in women 40 years and over which remains higher in NHW women) for all molecular subtypes and age brackets (with a specific focus on women under 40 years), the rate differences are similar or higher for NHB women. This means that reliance on family history guidelines for screening for earlier onset BC will differentially affect NHB women compared with NHW women.

### But what about the cost?

A key argument against population-based genetic testing has been resource related. However, simulation studies support the cost-effectiveness of population-based screening. Earlier studies have found population-based *BRCA 1/2* testing to be cost-effective in the Ashkenazi Jewish population^42,43^. Model simulations have now been extended to include the general population of women greater than 30 years old living in the UK, USA, Netherlands, China, Brazil, and India. In all countries but India, the results support that population-based, as opposed to family-based guidelines, are cost-effective from both a payer and societal perspective, the latter of which includes the benefits of decreased lives lost and increased life expectancy and work productivity^44^. For India, if the cost of genetic testing is further reduced from $200 (the model input) to $172 or $95 (which we can expect in the future), testing would also be cost-effective from a societal view and payer perspective view, respectively^44^. The cost-benefit estimates of these models are based on the advantages of identifying PV carriers for earlier risk reduction interventions, such as selective estrogen receptor modulators chemoprevention, risk-reducing mastectomy, and risk-reducing salpingo-oophorectomy^42–44^. The costs of these earlier risk reduction interventions are lower than the diagnosis and annual treatment costs if a woman were to develop breast or ovarian cancer^42–44^. While not specifically modeled, there is also the benefit of providing women with higher BC risk tailored interventions aimed towards modifiable lifestyle behaviors. Though the cost effectiveness simulations have focused on *BRCA1/2*, the reality is that in many settings, genetic testing for these genes are included on a larger panel of cancer susceptibility genes as well. These genes include, but are not limited to: ATM, BARD1, BRIP1, CDH1, CHEK2, MLH1, MSH2, PALB2, PMS2, PTEN, RAD51C, RAD51D TP53. Model simulations suggest such gene panels to be cost-effective for the general population, though more data is needed to recommend specific genes for multi-panel testing^45^.

### Challenges for implementation

By suggesting that we consider population-based testing as a priority, we cannot ignore the very real implementation challenges. We also acknowledge that even a change in criteria from family-based to testing all women diagnosed with TNBC and/or ovarian cancer has not removed differences in uptake of genetic testing across groups or removed health care system-level inequities^46^. In addition to family history, other commonly identified barriers to genetic testing include costs, privacy and confidentiality of results, clinical utility, and psychological harm^47^. While these barriers are often addressed by the clinician and/or genetic counselor, rate limiting is a significant difference by race in physician referral rates to genetic counseling^48,49^.

Population-based testing will demand more support from genetic counseling and implementation challenges are real. Extension to other BC susceptibility genes will also create additional complexities including the identification of variants of uncertain significance which may differ across groups^50^. We recognize the next essential steps would be to conduct implementation science studies to provide evidence relevant to how this could be done. However, these implementation challenges are true for all women, and continuing to rely primarily on family-based guidelines will translate into missing women who may benefit from earlier BC screening, especially NHB women, whom data support are disproportionately missed at a higher rate under current guidelines. Further, implementing population-based testing requires factors beyond just accessibility of genetic counselors and includes patient interest in testing. As indicated in Fig. 1, reliance on family-based guidelines also requires the role of many different providers in offering testing, which favors women connected to a health care system that is challenging to navigate for many and leads to fewer NHB women being offered genetic testing even though the prevalence of BC PV is similar between NHB women and NHW. Nonetheless, we believe that implementation concerns are greatly outweighed by the need for health equity given the greater BC morbidity and mortality burden born by NHB women. While these implementation challenges for population-based PV specific screening will require appreciable consideration, so do the reasons why we continue to implement family-based guidelines in the face of these unequal outcomes.

We also offer that the routine offering of multigene panel testing is a system-level factor whereas the uptake of counseling and genetic testing is an individual-level factor. For example, in an urban academic medical center, medical records were reviewed from 2016-2017 for women who were referred to a genetic counselor for hereditary breast and ovarian cancer^51^. The study found no racial difference in making a genetic counseling appointment or keeping the genetic counseling appointment. However, even in this study, only 53% of women offered counseling received it. Moreover, there is not a 1-to-1 ratio between genetic counseling and *receiving* genetic testing results. Halbert and colleagues have reported that only 47% of NHB women who received pre-test counseling for *BRCA1* and *BRCA2* mutations receive their results^33^. Hann and colleagues systematically reviewed 41 studies and found that black participants highly endorse statements about the benefits of genetic counseling and testing for cancer risk and endorse limitations to a lesser degree^52^. However, the review also highlights the ethical, legal, and social implications related to the awareness, knowledge, perception, and attitudes towards genetic testing. This includes, and is not limited to, variants of uncertain significance, and historical medical injustices. These factors have been, and will continue to be, examined across a range of racial and ethnic groups given the pivotal role they play in individual decision making. These are major considerations and barriers that we recognize. Therefore, in parallel, and not *in lieu* of, dual-efforts towards system-level offering of multigene panel testing and the implementation of inclusive and culturally sensitive cancer risk reduction programs that assist in individual-decision making are needed.

### Moving forward

We believe it is time to consider moving beyond family-based criteria for identifying higher risk women for earlier BC screening and targeted risk reduction strategies. Population-based genetic screening means we will no longer miss women who can benefit from earlier identification and intervention. What remains clear is that family history is more than just genes, and given the percent of misclassification between family history and known PV, approaches that screen all women for PV regardless of family history, but still have surveillance for women with family history but no known PV, may provide the highest sensitivity for detecting BC earlier.

The similarity in the prevalence of PVs in key BC susceptibility genes between NHB and NHW TNBC patients contrasts sharply to the doubling of incidence in NHB compared with NHW women, which means that while genes are critically important, so is research to understand the environmental factors causing this higher incidence in NHB women and that likely interact with underlying susceptibility. It is also important to note that the BC susceptibility genes we discuss are important not just for TNBC, but for all subtypes, and, thus, our appeal for population-based testing would benefit women at risk for BC irrespective of molecular subtype.

We propose that population-based genetic screening can help health providers identify women at higher risk earlier and, as a result, promote screening, and tailored risk reduction messages. To improve outcomes for all women and address the long-standing health inequities in the BC burden, we suggest rethinking complex family history-based and model-based guidelines and, instead, offering population-based genetic testing as one approach to reduce health inequities in receiving earlier BC screening and interventions.

### Reporting summary

Further information on research design is available in the Nature Research Reporting Summary linked to this article.

## Supplementary information

Supplemental Figure 1

reporting summary

## Data Availability

The datasets generated during and/or analyzed during the current study are available from the corresponding author on reasonable request.

## References

[1] Kehm RD, Yang W, Tehranifar P, Terry MB (2019). 40 years of change in age- and stage-specific cancer incidence rates in US women and men. JNCI Cancer Spectr..

[2] Lima SM, Kehm RD, Terry MB (2021). Global breast cancer incidence and mortality trends by region, age-groups, and fertility patterns. EClinicalMedicine.

[3] Acheampong T, Kehm RD, Terry MB, Argov EL, Tehranifar P (2020). Incidence trends of breast cancer molecular subtypes by age and race/ethnicity in the us from 2010 to 2016. JAMA Netw. Open.

[4] Mavaddat N (2012). Pathology of breast and ovarian cancers among BRCA1 and BRCA2 mutation carriers: results from the Consortium of Investigators of Modifiers of BRCA1/2 (CIMBA). Cancer Epidemiol. Biomark. Prev..

[5] Shimelis H (2018). Triple-negative breast cancer risk genes identified by multigene hereditary cancer panel testing. J. Natl. Cancer Inst..

[6] Bane AL (2007). BRCA2 mutation-associated breast cancers exhibit a distinguishing phenotype based on morphology and molecular profiles from tissue microarrays. Am. J. Surg. Pathol..

[7] Thompson T (2015). The context of collecting family health history: examining definitions of family and family communication about health among African American women. J. Health Commun..

[8] Hovick SR, Yamasaki JS, Burton-Chase AM, Peterson SK (2015). Patterns of family health history communication among older African American adults. J. Health Commun..

[9] Manswell Butty JA (2012). Evaluation findings from genetics and family health history community-based workshops for African Americans. J. Community Genet.

[10] Ademuyiwa FO (2019). Assessing the effectiveness of the National Comprehensive Cancer Network genetic testing guidelines in identifying African American breast cancer patients with deleterious genetic mutations. Breast Cancer Res Treat..

[11] National Comprehensive Cancer Network. *Genetic/Familial High-Risk Assessment: Breast, Ovarian, and Pancreatic (Version 1*.*2022**)*, https://www.nccn.org/professionals/physician_gls/pdf/genetics_bop.pdf.

[12] Beitsch PD (2019). Underdiagnosis of hereditary breast cancer: are genetic testing guidelines a tool or an obstacle?. J. Clin. Oncol..

[13] Yadav S (2020). Evaluation of germline genetic testing criteria in a hospital-based series of women with breast cancer. J. Clin. Oncol..

[14] Manahan ER (2019). Consensus guidelines on genetic‘ testing for hereditary breast cancer from the American society of breast surgeons. Ann. Surg. Oncol..

[15] Desai NV, Yadav S, Batalini F, Couch FJ, Tung NM (2021). Germline genetic testing in breast cancer: Rationale for the testing of all women diagnosed by the age of 60 years and for risk-based testing of those older than 60 years. Cancer.

[16] Pal T (2020). Points to consider: is there evidence to support BRCA1/2 and other inherited breast cancer genetic testing for all breast cancer patients? A statement of the American College of Medical Genetics and Genomics (ACMG). Genet Med.

[17] Sun, L. et al. A cost-effectiveness analysis of multigene testing for all patients with breast cancer. *JAMA Oncol*, 10.1001/jamaoncol.2019.3323 (2019). 10.1001/jamaoncol.2019.3323 PMC6777250 31580391

[18] Hu C (2021). A population-based study of genes previously implicated in breast cancer. N. Engl. J. Med.

[19] Gabai-Kapara E (2014). Population-based screening for breast and ovarian cancer risk due to BRCA1 and BRCA2. Proc. Natl. Acad. Sci. USA.

[20] Rowley SM (2019). Population-based genetic testing of asymptomatic women for breast and ovarian cancer susceptibility. Genet Med.

[21] Yadav S, Couch FJ (2019). Germline genetic testing for breast cancer risk: the past, present, and future. Am. Soc. Clin. Oncol. Educ. Book.

[22] Gudykunst WB, An LC (2001). agenda for studying ethnicity and family communication. J. Fam. Commun..

[23] RL, C. *Race and family: A structural approach*., (Sage Publications, 2006).

[24] Segrin C., F. J. *Family communication*. (Lawrence Erlbaum Associates, 2005).

[25] P., S. Who is kin? Family definition and African American families. *Journal of Human Behavior in the Social Environment* **15**, 163–181 (2007).

[26] Hull LE, Haas JS, Simon SR (2018). Provider discussions of genetic tests with U.S. women at risk for a BRCA mutation. Am. J. Prev. Med.

[27] Yao S (2012). Variants in the vitamin D pathway, serum levels of vitamin D, and estrogen receptor negative breast cancer among African-American women: a case-control study. Breast Cancer Res.

[28] Karliner LS (2007). Missed opportunities: family history and behavioral risk factors in breast cancer risk assessment among a multiethnic group of women. J. Gen. Intern Med.

[29] McCarthy AM (2016). Health care segregation, physician recommendation, and racial disparities in BRCA1/2 testing among women with breast cancer. J. Clin. Oncol..

[30] Armstrong J (2015). Utilization and outcomes of BRCA genetic testing and counseling in a national commercially insured population: the ABOUT study. JAMA Oncol..

[31] Halbert CH (2005). Recruiting African American women to participate in hereditary breast cancer research. J. Clin. Oncol..

[32] Olaya W (2009). Disparities in BRCA testing: when insurance coverage is not a barrier. Am. J. Surg..

[33] Halbert CH, Kessler L, Stopfer JE, Domchek S, Wileyto EP (2006). Low rates of acceptance of BRCA1 and BRCA2 test results among African American women at increased risk for hereditary breast-ovarian cancer. Genet Med.

[34] Manickam K (2018). Exome sequencing-based screening for BRCA1/2 expected pathogenic variants among adult biobank participants. JAMA Netw. Open.

[35] Manchanda R (2018). Current detection rates and time-to-detection of all identifiable BRCA carriers in the Greater London population. J. Med Genet.

[36] Domchek SM (2021). Comparison of the prevalence of pathogenic variants in cancer susceptibility genes in black women and non-hispanic white women with breast cancer in the United States. JAMA Oncol..

[37] Palmer, J. R. et al. Contribution of germline predisposition gene mutations to breast cancer risk in African American Women. *J Natl Cancer Inst*, 10.1093/jnci/djaa040 (2020). 10.1093/jnci/djaa040 PMC7735769 32427313

[38] McCarthy, A. M. & Armstrong, K. Genetic testing may help reduce breast cancer disparities for african american women. *J Natl Cancer Inst*, 10.1093/jnci/djaa042 (2020). 10.1093/jnci/djaa042 PMC7735768 32427327

[39] DeSantis CE (2019). Breast cancer statistics, 2019. CA Cancer J. Clin..

[40] Bethea TN (2016). Family history of cancer in relation to breast cancer subtypes in African American women. Cancer Epidemiol. Biomark. Prev..

[41] Couch FJ (2015). Inherited mutations in 17 breast cancer susceptibility genes among a large triple-negative breast cancer cohort unselected for family history of breast cancer. J. Clin. Oncol..

[42] Manchanda R (2015). Cost-effectiveness of population screening for BRCA mutations in Ashkenazi jewish women compared with family history-based testing. J. Natl. Cancer Inst..

[43] Manchanda R (2017). Cost-effectiveness of population based BRCA testing with varying Ashkenazi Jewish ancestry. Am. J. Obstet. Gynecol..

[44] Manchanda, R. et al. Economic evaluation of population-based BRCA1/BRCA2 mutation testing across multiple countries and health systems. *Cancers (Basel)* **12**, 10.3390/cancers12071929 (2020). 10.3390/cancers12071929 PMC7409094 32708835

[45] Manchanda R (2018). Cost-effectiveness of Population-Based BRCA1, BRCA2, RAD51C, RAD51D, BRIP1, PALB2 Mutation Testing in Unselected General Population Women. J. Natl. Cancer Inst..

[46] Kurian AW (2021). Time trends in receipt of germline genetic testing and results for women diagnosed with breast cancer or ovarian cancer, 2012–2019. J. Clin. Oncol..

[47] Smith-Uffen M, Bartley N, Davies G, Best M (2021). Motivations and barriers to pursue cancer genomic testing: a systematic review. Patient Educ. Couns..

[48] Peterson JM (2020). Racial disparities in breast cancer hereditary risk assessment referrals. J. Genet Couns..

[49] Chapman-Davis E (2021). Racial and ethnic disparities in genetic testing at a hereditary breast and ovarian cancer center. J. Gen. Intern Med.

[50] Yadav S (2021). Racial and ethnic differences in multigene hereditary cancer panel test results for women with breast cancer. J. Natl. Cancer Inst..

[51] Sutton AL (2020). Reducing disparities in receipt of genetic counseling for underserved women at risk of hereditary breast and ovarian cancer. J. Women’s Health (Larchmt.).

[52] Hann KEJ (2017). Awareness, knowledge, perceptions, and attitudes towards genetic testing for cancer risk among ethnic minority groups: a systematic review. BMC Public Health.

