# Supplementary material for: Do current family history-based genetic testing guidelines contribute to breast cancer health inequities?
Source: NPJ Breast Cancer. 2022 Mar 22;8:36. doi: 10.1038/s41523-022-00391-4 (PMC8941019; doi:10.1038/s41523-022-00391-4)
Supplement: Supplementary file 1 — Supplemental Figure 1 [file 41523_2022_391_MOESM1_ESM.pdf]

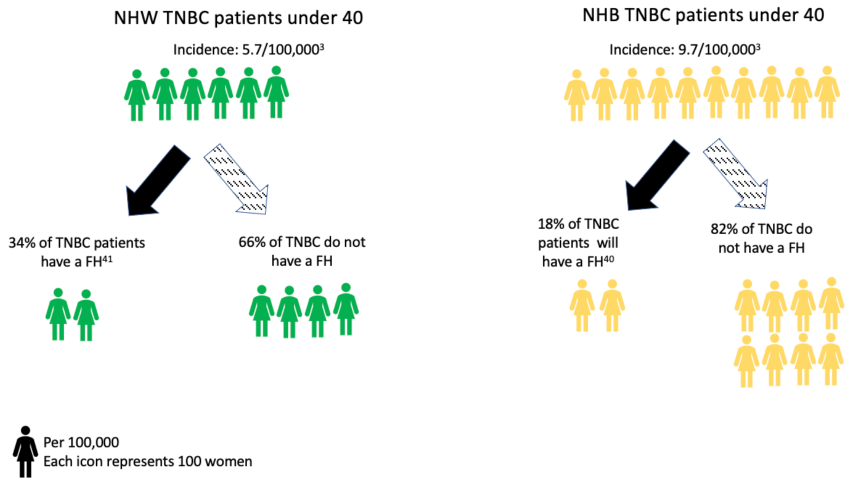

Supplemental Figure 1: Potential risk difference between NHB and NHW women with BC under 40 years old based on the estimated percent with family history of cancer. *Each female icon represents 100 women with TNBC. For women under 40, the incidences are 5.7 per 100,000 NHW women and 9.7 per 100,000 NHB women, respectively. Assuming that the percentages of women with FH are the same as for the age-adjusted numbers (34% of NHW TNBC patients report a family-history of BC and 18% of NHB TNBC patients report a family-history of BC), and assuming a conservative 100% uptake in genetic counseling and testing, this translates into an incidence rate difference between women under 40 with TNBC with a family history compared to women with TNBC without a family history of 8.0 versus 3.8 per 100,000 for NHB and NHW, respectively. Abbreviations: NHW, non-Hispanic white; NHB, non-Hispanic black; TNBC, triple negative breast cancer; FH, family history.*
